# Supplementary material for: Phosphatidylserine synthesis controls oncogenic B cell receptor signaling in B cell lymphoma
Source: J Cell Biol. 2023 Dec 4;223(2):e202212074. doi: 10.1083/jcb.202212074 (PMC10694799; doi:10.1083/jcb.202212074)
Supplement: Table S1 — lists human cancer cell lines used in this study. [file JCB_202212074_TableS1.docx]

| NAME | CELL  ORIGIN | CONDITION | CULTURE MEDIUM | RRID  ACCESSION |
| --- | --- | --- | --- | --- |
| RS4;11 | B-ALL | Suspension | RPMI1640 | CVCL_0093 |
| NALM-6 | B-ALL | Suspension | RPMI1640 | CVCL_0092 |
| Ramos | Burkitt’s lymphoma | Suspension | RPMI1640 | CVCL_0597 |
| Jeko-1 | Mantle cell lymphoma | Suspension | RPMI1640 | CVCL_1865 |
| SU-DHL-6 | GCB-DLBCL | Suspension | RPMI1640 | CVCL_2206 |
| SU-DHL-2 | ABC-DLBCL | Suspension | RPMI1640 | CVCL_9550 |
| MM.1S | Plasma cell myeloma | Suspension | RPMI1640 | CVCL_8792 |
| Jurkat | T-ALL | Suspension | RPMI1640 | CVCL_0065 |
| SNB-78 | Glioblastoma | Adherent | DMEM | CVCL_B321 |
| SNB-75 | Glioblastoma | Adherent | DMEM | CVCL_1706 |
| SF539 | Glioblastoma | Adherent | DMEM | CVCL_1691 |
| SF295 | Glioblastoma | Adherent | DMEM | CVCL_1690 |
| SF268 | Glioblastoma | Adherent | DMEM | CVCL_1689 |
| U-251 | Glioblastoma | Adherent | DMEM | CVCL_0021 |
| SK-OV-3 | Ovarian cancer | Adherent | DMEM | CVCL_0532 |
| OVCAR8 | Ovarian cancer | Adherent | DMEM | CVCL_1629 |
| OVCAR3 | Ovarian cancer | Adherent | DMEM | CVCL_0465 |
| DMS273 | Small cell lung cancer | Adherent | DMEM | CVCL_1176 |
| DMS114 | Small cell lung cancer | Adherent | DMEM | CVCL_1174 |
| NCI-H460 | Non-small cell lung cancer | Adherent | DMEM | CVCL_0459 |
| NCI-H226 | Non-small cell lung cancer | Adherent | DMEM | CVCL_1544 |
| NCI-H23 | Non-small cell lung cancer | Adherent | DMEM | CVCL_1547 |
| A549 | Non-small cell lung cancer | Adherent | DMEM | CVCL_0023 |
| HBC-5 | Breast cancer | Adherent | DMEM | CVCL_B497 |
| BSY-1 | Breast cancer | Adherent | DMEM | CVCL_B499 |
| HBC-4 | Breast cancer | Adherent | DMEM | CVCL_B496 |
| HCC1419 | Breast cancer | Adherent | DMEM | CVCL_1251 |
| SK-BR-3 | Breast cancer | Adherent | DMEM | CVCL_0033 |
| MCF-7 | Breast cancer | Adherent | DMEM | CVCL_0031 |
| MDA-MB-231 | Breast cancer | Adherent | DMEM | CVCL_0062 |
| HCT-15 | Colon cancer | Adherent | DMEM | CVCL_0292 |
| HT-29 | Colon cancer | Adherent | DMEM | CVCL_0320 |
| KM12 | Colon cancer | Adherent | DMEM | CVCL_1331 |
| HCT116 | Colon cancer | Adherent | DMEM | CVCL_0291 |
| HCC2998 | Colon cancer | Adherent | DMEM | CVCL_1266 |
| SW480 | Colon cancer | Adherent | DMEM | CVCL_0546 |
| SW620 | Colon cancer | Adherent | DMEM | CVCL_0547 |
| MKN74 | Stomach cancer | Adherent | DMEM | CVCL_2791 |
| MKN45 | Stomach cancer | Adherent | DMEM | CVCL_0434 |
| MKN28 | Stomach cancer | Adherent | DMEM | CVCL_1416 |
| MKN1 | Stomach cancer | Adherent | DMEM | CVCL_1415 |
| St-4 | Stomach cancer | Adherent | DMEM | CVCL_H265 |
| DU145 | Prostate cancer | Adherent | DMEM | CVCL_0105 |
| LOX-IMVI | Melanoma | Adherent | DMEM | CVCL_1381 |
| RXF631L | Renal cancer | Adherent | DMEM | CVCL_A780 |
| A431 | Epidermoid carcinoma | Adherent | DMEM | CVCL_0037 |
| HeLa | Cervical cancer | Adherent | DMEM | CVCL_0030 |

**Supplementary Table S1. Human cancer cell lines used in this study**
