# Supplementary material for: Phosphatidylserine synthesis controls oncogenic B cell receptor signaling in B cell lymphoma
Source: J Cell Biol. 2023 Dec 4;223(2):e202212074. doi: 10.1083/jcb.202212074 (PMC10694799; doi:10.1083/jcb.202212074)
Supplement: Table S2 — lists oligonucleotides used in this study. [file JCB_202212074_TableS2.docx]

| NAME | SEQUENCES (5’ to 3’) | APPLICATIONS |
| --- | --- | --- |
| sgPTDSS1_1F | CACCGACTCTTCCTGAATTTCGAGC | px458  gene editing |
| sgPTDSS1_1R | AAACGCTCGAAATTCAGGAAGAGTC |  |
| sgPTDSS1_2F | CACCGTCATGTACTTCGCCTTTACC |  |
| sgPTDSS1_2R | AAACGGTAAAGGCGAAGTACATGAC |  |
| sgPTDSS2_1F | CACCGGCAGGGACGCCGGAGGTCCG |  |
| sgPTDSS2_1R | AAACCGGACCTCCGGCGTCCCTGCC |  |
| sgPTDSS2_2F | CACCGTGGCCGGCAGACGGCCCGTC |  |
| sgPTDSS2_2R | AAACGACGGGCCGTCTGCCGGCCAC |  |
| sgCD79B_1F | CACCGAGTACCAGCAGCCAGATCGG |  |
| sgCD79B_1R | AAACCCGATCTGGCTGCTGGTACTC |  |
| sgCD79B_2F | CACCGGTAGTGCTTGTTCGCGGATC |  |
| sgCD79B_2R | AAACGATCCGCGAACAAGCACTACC |  |
| sgCD19_1F | CACCGAAGCGGGGACTCCCGAGACC |  |
| sgCD19_1R | AAACGGTCTCGGGAGTCCCCGCTTC |  |
| sgCD19_2F | CACCGTGGAATGTTTCGGACCTAGG |  |
| sgCD19_2R | AAACCCTAGGTCCGAAACATTCCAC |  |
| sgCD22_1F | CACCGAAACCCTCTACGCCTGGGAG |  |
| sgCD22_1R | AAACCTCCCAGGCGTAGAGGGTTTC |  |
| sgCD22_2F | CACCGGGGAGAACTTGAGCTCGCTC |  |
| sgCD22_2R | AAACGAGCGAGCTCAAGTTCTCCCC |  |
| sgOSBPL5_F | CACCGGGTGCTACCCACGTTCGTAC |  |
| sgOSBPL5_R | AAACGTACGAACGTGGGTAGCACCC |  |
| sgOSBPL8_F | CACCGTGCAAATCTTTGGTTGGCGT |  |
| sgOSBPL8_R | AAACACGCCAACCAAAGATTTGCAC |  |
| sgPITPNM1_F | CACCGCTACTGGGAGTCCGCCGACG |  |
| sgPITPNM1_R | AAACCGTCGGCGGACTCCCAGTAGC |  |
| sgPITPNM2_F | CACCGCGTCAAGCACGAAGCCGTCT |  |
| sgPITPNM2_R | AAACAGACGGCTTCGTGCTTGACGC |  |
| YC3.6_F | GACCATCCTCTAGACTGCCGGAGACCCAAGCTTGCGGC | Plasmid  construction |
| YC3.6_R | CGGCCCTCGAGGCCTGCAGGGATGGATATCTGCAGAATTC |  |
| EGFP-2xP4M^SidM^_F | TTAGTGAACCGTCAGATCCGCACCATGGTGAGCAAGGG |  |
| EGFP-2xP4M^SidM^_R | TTGATCCCTCGATGTTAACTTTATTTTATCTTAATGGTTTGTCTTTCTTGAGAC |  |
| PH^Akt^-EGFP_F | TTAGTGAACCGTCAGATCCGCACCATGAGCGACGTGGC |  |
| PH^Akt^-EGFP_R | TTGATCCCTCGATGTTAACTTTACTTGTACAGCTCGTCCATGC |  |
| mCherry-ORP5_F | TTAGTGAACCGTCAGATCCGCACCATGGTGAGCAAGGGCGAG |  |
| mCherry-ORP5_R | TTGATCCCTCGATGTTAACTCTATTTGAGGATGTGGTTAATGAACAG |  |
| mCherry-ORP8_F | TTAGTGAACCGTCAGATCCGCACCATGGTGAGCAAGGGCGAG |  |
| mCherry-ORP8_R | TTGATCCCTCGATGTTAACTCTACTTGAACATGAAGTTTATTATGACTTG |  |
| Nir2-EGFP_F | TGGTTTAGTGAACCGTCAGATCCGCACCATGCTCATCAAGGAATACCACATTC |  |
| Nir2-EGFP_R | TTGCTCACCGACCCACCTCCGCCTGACTCCTCGCTGTCCAGCTTC |  |
| Nir3-EGFP_F | TGGTTTAGTGAACCGTCAGATCCGCACCATGATTATAAAGGAATATCGGATTCCTCTGCCAATGACCGTG |  |
| Nir3-EGFP_R | TTGCTCACCGACCCACCTCCGCCTGACTTGGGGCCCGCGGCTGC |  |
| PI4KIIα_F | TGGTTTAGTGAACCGTCAGATCCGCACCATGGACGAGACG |  |
| PI4KIIα_R | TTGCTCACCGACCCACCTCCGCCTGACCACCATGAAAAGA |  |
| PI4KIIβ_F | TGGTTTAGTGAACCGTCAGATCCGCACCATGGAGGATCCC |  |
| PI4KIIβ_R | TTGCTCACCGACCCACCTCCGCCTGACCAGGAGGAAAAAA |  |
| PI4KIIIα_F | TTGCTCACCGACCCACCTCCGCCTGAGTAGGGGATGTCAT |  |
| PI4KIIIα_R | TGGTTTAGTGAACCGTCAGATCCGGCGGCTCGTGAGGTGA |  |
| PI4KIIIβ_F | TGGTTTAGTGAACCGTCAGATCCGCACCATGAGATTCTTG |  |
| PI4KIIIβ_R | TTGCTCACCGACCCACCTCCGCCTGACATGATGCCGTTGG |  |
| SACM1L_F | TGGTTTAGTGAACCGTCAGATCCGCACCATGGCGACGGCGGCCTACGAG |  |
| SACM1L_R | TTGCTCACCGACCCACCTCCGCCTGAGTCTATCTTTTCTTTCTGGACCAGTCTGGGAG |  |
| GFP-MAPPER_F | TGGTTTAGTGAACCGTCAGATCCGCACCATGGATGTATGCGTCCGTC |  |
| GFP-MAPPER_R | AAGCTTGATCCCTCGATGTTAACTTCAAGTTACTGAATCTTTCTTCTTCCGGAATG |  |
| hPI4K2A_F | AAGCAGAACCTCTTCCTGAGAAC | qRT-PCR |
| hPI4K2A_R | TCCTTCACCACCACCCAGTC |  |
| hPI4K2B_F | GCTGAGAGCAGAGCAAGCAA |  |
| hPI4K2B_R | AAAGCAGCAAGGGCAGCA |  |
| hPI4KA_F | GCTGACGGATGAGATGGTGATG |  |
| hPI4KA_R | GAGAGTGACCAGGGAGACGA |  |
| hPI4KB_F | CAGCCTTCAAACGAGACCCA |  |
| hPI4KB_R | CTCTTGCCGAAGGTCATCCC |  |
| hPIP4K2A_F | CCTGGCGAGCAAGACCAAGA |  |
| hPIP4K2A_R | ATCTGGCATCAACATAACAGGGAT |  |
| hPIP4K2B_F | CGTGTGCCAGAAAGTGAAGC |  |
| hPIP4K2B_R | GCTGGGCAGGTTCTCCTTATT |  |
| hPIP4K2C_F | GTTTCGGCTTCGCCTCCAA |  |
| hPIP4K2C_R | CTTGATCTTGGAGCTGGCCTTA |  |
| hPIP5K1A_F | TCATTGGTGCCTTATGCCTCTG |  |
| hPIP5K1A_R | AGGACATCACGCTCTGGTTTG |  |
| hPIP5K1B_F | ACTGGTCTTGGTAGCGGAGG |  |
| hPIP5K1B_R | GGCACCAGAGAAAGGTCACAG |  |
| hPIP5K1C_F | TTTGGCTCAGAAGAAGGCGG |  |
| hPIP5K1C_R | GAGGTGGTCTTCTTGTAGGTGG |  |
| hPIK3CD_F | CATATGTGCTGGGCATTGGC |  |
| hPIK3CD_R | TTTCACAGTAGCCCCGGAAC |  |
| hSACM1L_F | CAGAGCTGGTGTGCGCTATT |  |
| hSACM1L_R | TTGCTGATCTGTGGCAGTGG |  |
| hINPP5F_F | CCAAGGACCACTACATCCTGC |  |
| hINPP5F_R | ACCAACCCCAAACAAATGGGA |  |
| hFIG4_F | CCATCATCAGCTCGGTCCAG |  |
| hFIG4_R | ATCCAAGCGGCCAAGAAGTT |  |
| hSYNJ1_F | GGTGGGCGTCTGTTTGTTTG |  |
| hSYNJ1_R | GACGAAGCAAAGGCTGGTTG |  |
| hSYNJ2_F | ACGACTGCCTGCTGTTCGAG |  |
| hSYNJ2_R | GCCACCAGATTTCAGCCTCA |  |
| hOCRL_F | TCGGGCTGAACCTTTTGAGG |  |
| hOCRL_R | TCGATCCAGGTGAAGGACGA |  |
| hINPP5D_F | CAACAGCAAACCGGGGTCTC |  |
| hINPP5D_R | TTCCTGGGAGCAGGCTTAGG |  |
| hINPP5E_F | TACGACAGCACCTCCAAGCA |  |
| hINPP5E_R | AGCTGCCAACGGAATGTTGTCT |  |
| hINPP5K_F | CCGCCCACCTACAAGTTTGA |  |
| hINPP5K_R | ACAAGGAGAAGTGTGACGCC |  |
| hINPP5B_F | GACATGGTTCGCTCCTCCAC |  |
| hINPP5B_R | ACAGCCGGAGGCATTCTTTG |  |
| hINPP5J_F | GAGTTCGGTGAGGATGCAGG |  |
| hINPP5J_R | GTGGCAGTTCAGGAAGCAGAG |  |
| hSHIP2_F | GCACGAGAACCGTATCAGCC |  |
| hSHIP2_R | GCCGCAGGATGTCCAAGTAG |  |
| hTMEM55A_F | CATTCCTGTGGATGGAACTGAGG |  |
| hTMEM55A_R | GGTTGCTCGAAATCGCCTTG |  |
| hTMEM55B_F | GTACGGAGCCGGTAAACATGCC |  |
| hTMEM55B_R | ACGTTGATGAGAGATTGGCAGAC |  |
| hPTEN_F | CCACACGACGGGAAGACAAG |  |
| hPTEN_R | AGGTTTCCTCTGGTCCTGGTATG |  |
| hOSBPL5_F | GTGCCGCTGGAGGAGCAGAC |  |
| hOSBPL5_R | AGGGGCTGTGGTCCTCGTATC |  |
| hOSBPL8_F | GAACAGGGAGATTTTGAATCA |  |
| hOSBPL8_R | TCCTGTGAGTGGATCAAGTTC |  |
| hPITPNM1_F | CTGGAAGGGCAGTGAGTTGG |  |
| hPITPNM1_R | CAGTAGCTGGCGTGGAAGAG |  |
| hPITPNM2_F | GCTGAGAGCTACACGGCATC |  |
| hPITPNM2_R | CAATGTCCAGCTCAGGGAGG |  |
| h36B4_F | GTGTTCGACAATGGCAGCAT |  |
| H36B4_R | GACACCCTCCAGGAAGCGA |  |

**Supplementary Table S2. Oligonucleotides used in this study**
