# Supplementary figures and images for: Phosphatidylserine synthesis controls oncogenic B cell receptor signaling in B cell lymphoma
Source: J Cell Biol. 2023 Dec 4;223(2):e202212074. doi: 10.1083/jcb.202212074 (PMC10694799; doi:10.1083/jcb.202212074)

S2A

IB: anti-PTDSS1

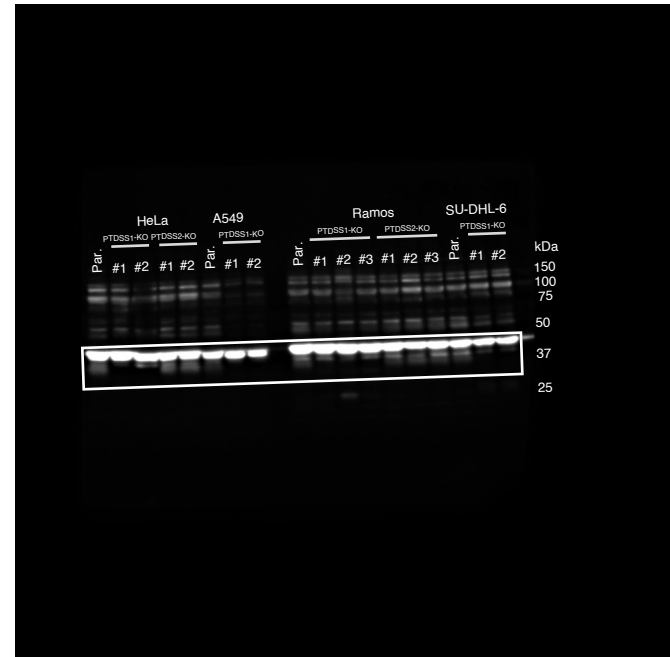

IB: anti-PTDSS2

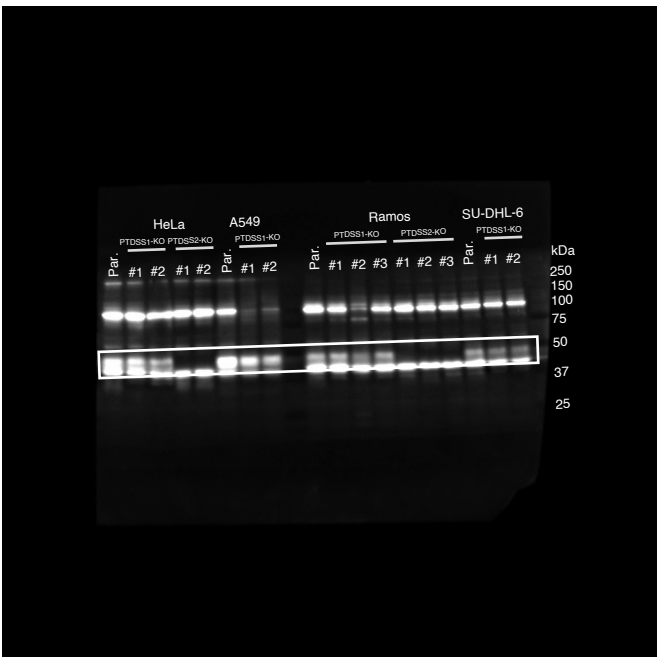

IB: anti- $\alpha$ -tubulin

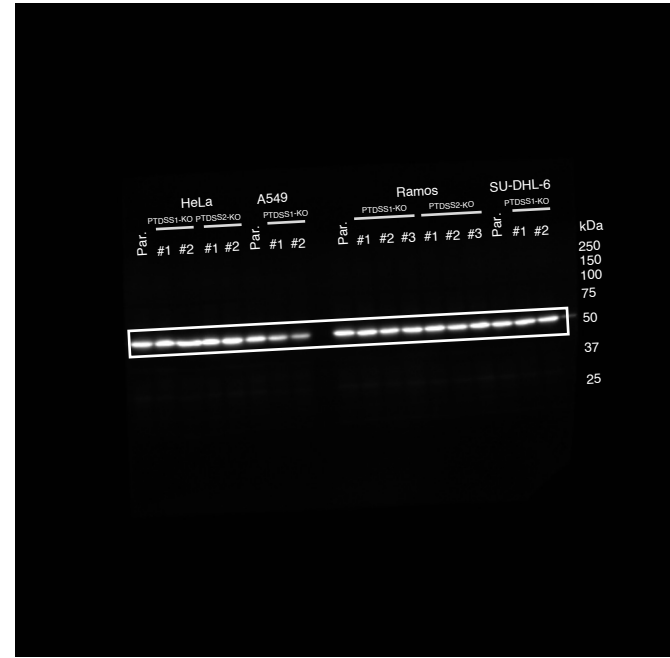

Supplement: SourceData FS2 — is the source file for Fig. S2. [file JCB_202212074_SourceDataFS2.pdf]

S5B

IB: α-ORP8

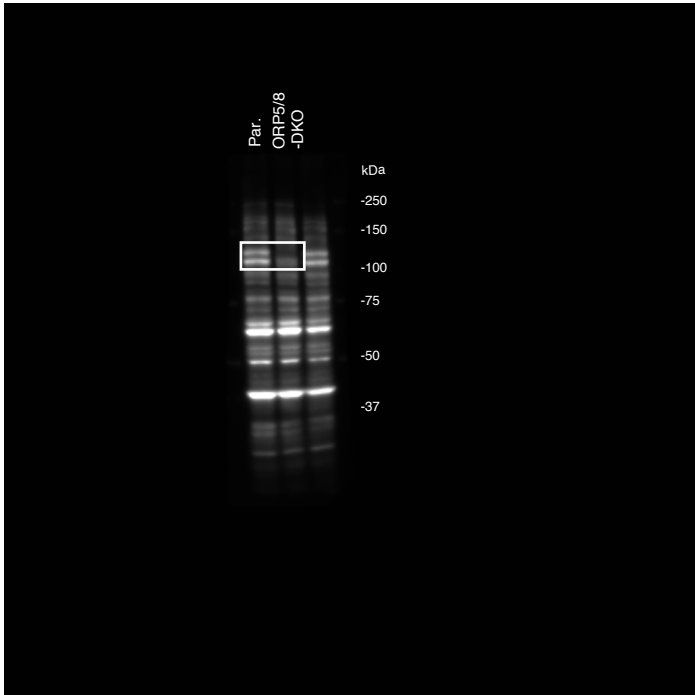

IB: anti-α-tubulin

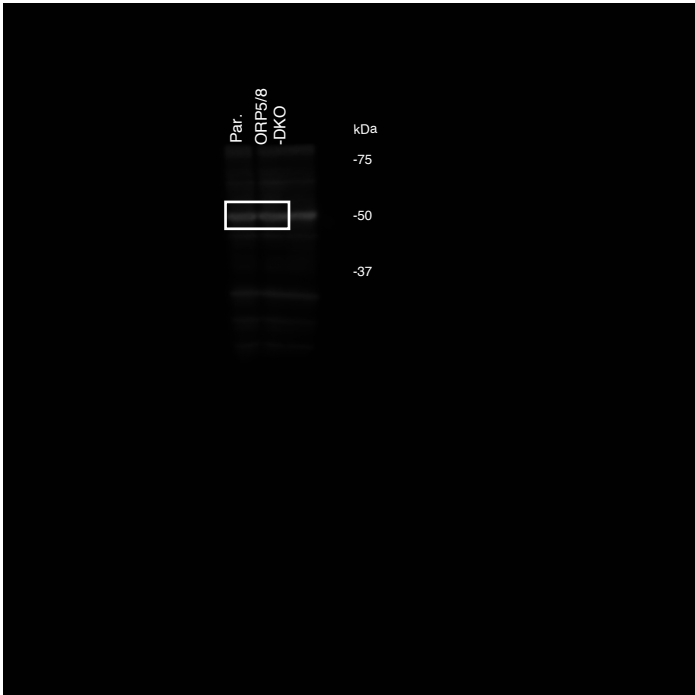

S5C

IB: α-Nir2

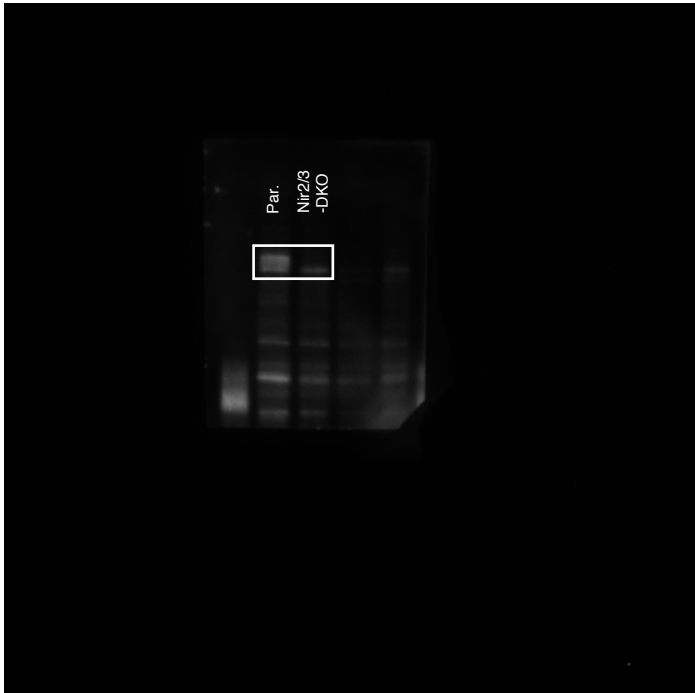

IB: anti-α-tubulin

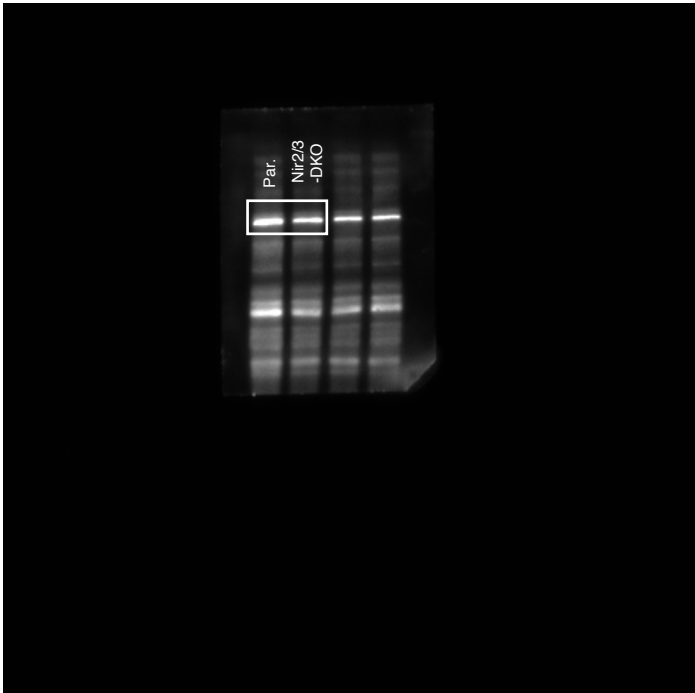

Supplement: SourceData FS5 — is the source file for Fig. S5. [file JCB_202212074_SourceDataFS5.pdf]
